# Re-Evaluation of Sarcolemma Injury and Muscle Swelling in Human Skeletal Muscles after Eccentric Exercise

**DOI:** 10.1371/journal.pone.0062056

**Published:** 2013-04-15

**Authors:** Ji-Guo Yu, Jing-Xia Liu, Lena Carlsson, Lars-Eric Thornell, Per S. Stål

**Affiliations:** 1 Department of Surgical and Perioperative Sciences, Sports Medicine Unit, Umeå University, Umeå, Sweden; 2 Department of Integrative Medical Biology, Section for Anatomy, Umeå University, Umeå, Sweden; University of Canberra, Australia

## Abstract

The results regarding the effects of unaccustomed eccentric exercise on muscle tissue are often conflicting and the aetiology of delayed onset muscle soreness (DOMS) induced by eccentric exercise is still unclear. This study aimed to re-evaluate the paradigm of muscular alterations with regard to muscle sarcolemma integrity and fibre swelling in human muscles after voluntary eccentric exercise leading to DOMS. Ten young males performed eccentric exercise by downstairs running. Biopsies from the soleus muscle were obtained from 6 non-exercising controls, 4 exercised subjects within 1 hour and 6 exercised subjects at 2–3 days and 7–8 days after the exercise. Muscle fibre sarcolemma integrity, infiltration of inflammatory cells and changes in fibre size and fibre phenotype composition as well as capillary supply were examined with specific antibodies using enzyme histochemistry and immunohistochemistry. Although all exercised subjects experienced DOMS which peaked between 1.5 to 2.5 days post exercise, no significant sarcolemma injury or inflammation was detected in any post exercise group. The results do not support the prevailing hypothesis that eccentric exercise causes an initial sarcolemma injury which leads to subsequent inflammation after eccentric exercise. The fibre size was 24% larger at 7–8 days than at 2–3 days post exercise (p<0.05). In contrast, the value of capillary number per fibre area tended to decrease from 2–3 days to 7–8 days post exercise (lower in 5 of the 6 subjects at 7–8 days than at 2–3 days; p<0.05). Thus, the increased fibre size at 7–8 days post exercise was interpreted to reflect fibre swelling. Because the fibre swelling did not appear at the time that DOMS peaked (between 1.5 to 2.5 days post exercise), we concluded that fibre swelling in the soleus muscle is not directly associated with the symptom of DOMS.

## Introduction

The aetiology of delayed onset muscle soreness (DOMS) induced by eccentric exercise (i.e., lengthening of a contracting muscle) is still unclear [1]. The prevailing hypothesis of the mechanisms of DOMS is that the excessive strain produced during eccentric muscular contraction induces extracellular or intracellular membrane disruption that may induce hydrolysis of structural proteins such as desmin intermediate filament network, leading to myofibrillar disorganization in the form of Z-band streaming or complete disruption [2], [3]. This is followed by fibre necrosis and inflammatory cell infiltration that potentiate the nerve endings and perception of pain. Passive manipulation and active movement alter intramuscular pressure and stimulate mechanoreceptor nerve endings, contributing to the perception of soreness [4]. However, recent studies in humans provided evidence which did not support the hypothesis, e.g., voluntary eccentric exercise did not lead to desmin intermediate filament network hydrolysis [5], [6], myofibrillar disruption [5], [7] or muscle fibre inflammation and necrosis [5], [6], [8], [9], [10]. The Z-band streaming, classically proposed to be a hallmark of muscle damage after eccentric exercise [11], [12] was also proved to represent myofibril remodelling [7], [13], [14], [15], [16].

As proposed in the prevailing hypothesis, sarcolemma damage has indeed been observed in animals soon after electrical stimulation-induced eccentric muscular contractions, and the damage was often amplified later [17], [18], [19]. In contrast, studies on humans after voluntary eccentric exercise revealed no [5], [6], [20], [21], [22], [23] or only minor damage [10], [24] in muscle fibre membrane. To our knowledge, no data is available in humans with regard to the initial reactions of muscle fibre membrane to acute voluntary eccentric exercise.

Eccentric exercise has been proposed to cause intracellular fibre swelling which is associated with the subsequent muscle soreness and stiffness [3], [4], [25], [26]. Because of technique limitations in directly measuring intracellular fibre swelling, many studies measured muscle swelling using either sonography or circumference of the exercised extremity. Although many studies have reported muscle swelling in human muscles after voluntary eccentric exercise [27], [28], [29], none have shown a time course correlation between muscle swelling and DOMS. By measuring muscle fibre size and intracellular pressure, two studies observed fibre swelling after voluntary eccentric exercise [25], [30]; however, the studies only analysed muscle biopsies taken 2 days after exercise and therefore there is no information on how fibre swelling develops in relation to the time course of DOMS after eccentric exercise.

In skeletal muscle the capillary network is very dynamic and general exercise can cause adaptive changes in capillary supply [31]. In human muscles a single bout of intensive exercise has been shown to up-regulate angiogenic factors [32], whereas in animals a few days of electrical stimulation induced increased capillarization [33]. Electrical stimulation-induced eccentric exercise and downhill running have been reported to cause morphological changes in capillary luminal area and microvascular dysfunction in rat skeletal muscle [34], [35]. To date it is still unclear whether voluntary eccentric exercise in humans is also harmful to the microvascular system.

By analyzing muscle biopsies taken from human soleus muscle within 1 hour, at 2–3 days and 7–8 days after downstairs running, the present study aimed to reveal changes in 1) muscle fibre sarcolemma integrity; 2) muscle fibre swelling/fibre size; 3) microvascular system; and finally 4) to examine the time course of these changes (if any) in relation to the development of DOMS.

## Materials and Methods

### Ethics Statement

All participants were informed about the design of the study and were asked to refrain from unaccustomed exercise during the experimental period. Written informed consent was obtained from all participants. The study protocol was approved by the Ethics Committee, Medical Faculty, Umeå University.

### Subjects

Sixteen healthy males (mean age 24 years, range 21–30) participated in the study. The subjects were young and healthy sedentary medical students, and none of them were accustomed to eccentric exercise or involved in any regular exercise regime. All the subjects were asked to refrain from any strenuous physical exercise before the experiment. Ten of the subjects were randomly assigned to exercise group and the rest served as controls.

### Experimental Procedures

In brief, the subjects were asked to run downstairs from the 10th floor to the ground floor and then took the elevator back to the 10th floor, and to repeat the procedure 15 times. During the downstairs running, the subjects were asked to use the ball of the foot to touch the ground to support the body mass and then to the full foot. By doing so, the soleus muscle was subjected to eccentric contraction.

### Muscle Soreness Evaluation and Muscle Biopsy

Evaluation of DOMS in the soleus muscle of all subjects was described in our previous work [14]. In brief, muscle soreness was self-evaluated over the middle of the soleus muscles with the muscle contracted (loaded) and relaxed (unloaded). The muscle soreness was rated by the subjects before and after the exercise twice daily for 8.5 days on a 0–10 subject rating scale (0 = no soreness and 10 = very, very sore).

Muscle biopsies were taken from the lateral border of the middle of the soleus muscle (accessible through contraction of the triceps surae). Biopsies from the soleus muscle were obtained under local anaesthesia from 6 controls, 4 exercised subjects within 1 hour (1 h group) and 6 exercised subjects at 2–3 days (2–3 day group) and 7–8 days (7–8 day group; the contralateral leg) after the exercise. The muscle biopsy was mounted in embedding medium (Tissue Tek®, O.C.T. Compound, Miles laboratories, Naperville, IL, USA), frozen in propane chilled with liquid N_2_ (−160 °C) and stored at −80 °C until used.

### Histochemistry and Immunohistochemistry (IHC)

Serial muscle cross-sections were cut at −25 °C by using a Reichert Jung cryostat (Leica, Nussloch, Germany). Eight µm thick sections were stained with haematoxylin-eosin and a modified Gomori trichrome staining for basic histopathology including detection of degenerative processes and inflammation [36].

Five µm thick sections were processed for IHC with different previously characterized antibodies. For fibre phenotype type classification, serial cross sections were stained with monoclonal antibodies (mAbs) against different adult and developmental myosin heavy chain (MyHC) isoforms [37]: A4.840 (strong affinity for MyHCI), A4.74 (strong affinity for MyHCIIa), N2.261 (strong affinity to MyHCIIa, weak affinity to MyHCI, no affinity to MyHCIIx), BF-35 (strong affinity for all MyHC isoforms except IIx), F1.652 (strong affinity to embryonic MyHC) and NCL-MHCn (strong affinity to fetal MyHC). All antibodies, except NCL-MHCn, were obtained from the Developmental Studies Hybridoma Bank, developed under the auspices of the NICHD and maintained by the University of Biological Sciences, Iowa City, Iowa, USA. NCL-MHCn was obtained from Novocastra Lab, Newcastle, UK.

A panel of antibodies was used for visualization of muscle fibre membrane integrity. Monoclonal Abs Dys1, Dys2, Dys3 (Novocastra laboratories, Newcastle upon Tyne, UK) and polyclonal antibody 5EM96 (gift from Dr. S. C. Watkins, CBI, Pittsburgh, PA, USA) stains muscle fibre plasma membrane protein dystrophin, and mAb 5H2 against laminin α2-chain (Novocastra Lab, Newcastle, UK) stains the basement membranes of muscle fibres. Muscle fibre membrane integrity was also evaluated through identification of cellular distribution of plasma proteins fibronectin and fibrinogen. Fibronectin has been previously used as an indicator for membrane injury in muscle fibre [18] and fibrinogen as a sensitive marker for membrane damage in cardiomyocytes after myocardial infarction [38]. Myocardial tissue samples obtained from experimental myocardial infarction in pigs were used as control for specificity of the staining reaction. MAb 52BF12 was used for plasma protein fibronectin [39]. Three different antibodies against plasma protein fibrinogen were used: mAb F 9902 (Sigma, Copenhagen, Denmark), polyclonal Ab A0080 (Dako, Carpinteria, USA) and polyclonal Ab Pc 056 (The Binding site Inc., San Diego, CA, USA).

Identification of capillaries was performed with mAb 4C7 against laminin α5-chain in the capillary basement membrane (Chemicon, Temecula, Calif., USA) [40] and Ulex europaeus agglutin 1 lectin (UEA-1, Dako, Glostrup, Denmark), an endothelial cell marker that previously has been shown to specifically recognize capillaries in skeletal muscle. The mAb 4C7 labels the basement membrane of capillaries strongly and the basement membrane of muscle fibre weakly [39]. To identify monocytes/macrophages, mAb F7135 (Dako, Glostrup, Denmark) against CD68 was used [41].

The IHC staining follows the same procedures as described previously [14]. Visualization of bound primary antibodies was performed by indirect fluorescence, using Alexa 488 (green) or Alexa 568 (red) conjugated secondary antibodies (Molecular Probes Inc., Eugene, USA) [14], or by indirect peroxidase-antiperoxidase (PAP) staining [37]. Control sections were treated as above, except that the primary antibody was exchanged with non-immune serum.

### Sarcolemma Integrity

Analysis of the muscle sarcolemma integrity was performed on sections stained with antibodies against dystrophin, laminin α2-chain, fibronectin and fibrinogen. From each muscle sample randomly chosen areas covering a minimum of 300 fibres were scanned and photographed using a Nikon microscope (Eclipse, E800, Tokyo, Japan) equipped with a Spot RT camera (Diagnostic Instrument, Sterling Heights, USA). The sections stained with the antibodies against dystrophin and laminin α2-chain were examined for disruption or absence of staining from membrane, whereas the sections stained with antibodies against plasma proteins were examined for intracellular staining outlined by double labelling of dystrophin or laminin. The number of fibres with visible lack of staining on membrane or intracellular staining was counted separately and expressed as percentage of total fibres in the photographed area.

### Morphometric Analysis

For estimation of muscle fibre size, fibre type classification and capillary network, four randomly chosen areas in each of the sections stained with antibodies against laminin α2-chain, UEA-I lectin and sections double stained for α5-chains and different MyHC isoforms were scanned and photographed with a MTI CCD 72 video camera (DAGE-MTI, Michigan City, USA) through a Zeiss microscope (Axiophot, Carl Zeiss, Oberkochen, Germany) equipped with an image analysis system (IBAS, Kontron Elektronik GMBH, Eching, Germany).

To estimate fibre size and capillary supply, the circumference of each fibre and capillary was traced on digital image along the periphery of the basement membrane revealed by staining for laminin α5-chain and UEA-I lectin. The IBAS program calculated the area of each individual muscle fibre in the muscle fibre cross-section. Fibre size variability as coefficient of variation (CV) was estimated according to the formula CV = (SD/mean fibre diameter) x 100%. Capillary density (CD) was calculated as the total number of capillary per mm^2^ muscle cross-section and capillary per fibre (C/F) was expressed as the ratio CD/number of fibre per mm^2^ cross-sectional tissue. For the analysis of the number of capillaries around each individual fibre (CAF), all capillaries within a distance of 5 µm from each muscle fibre were included. The variable of CAF related to the cross-sectional area of each individual fibre (CAFA) was calculated according to the formula: CAF/fibre cross-sectional area x 10^3^. On average, 349 fibres per sample (totally 6821 fibres) were individually analysed for capillary supply, which is beyond the reported requirement (50 fibres/sample) for a reliable analysis of the capillary network in normal limb muscles [42].

**Figure 1:**
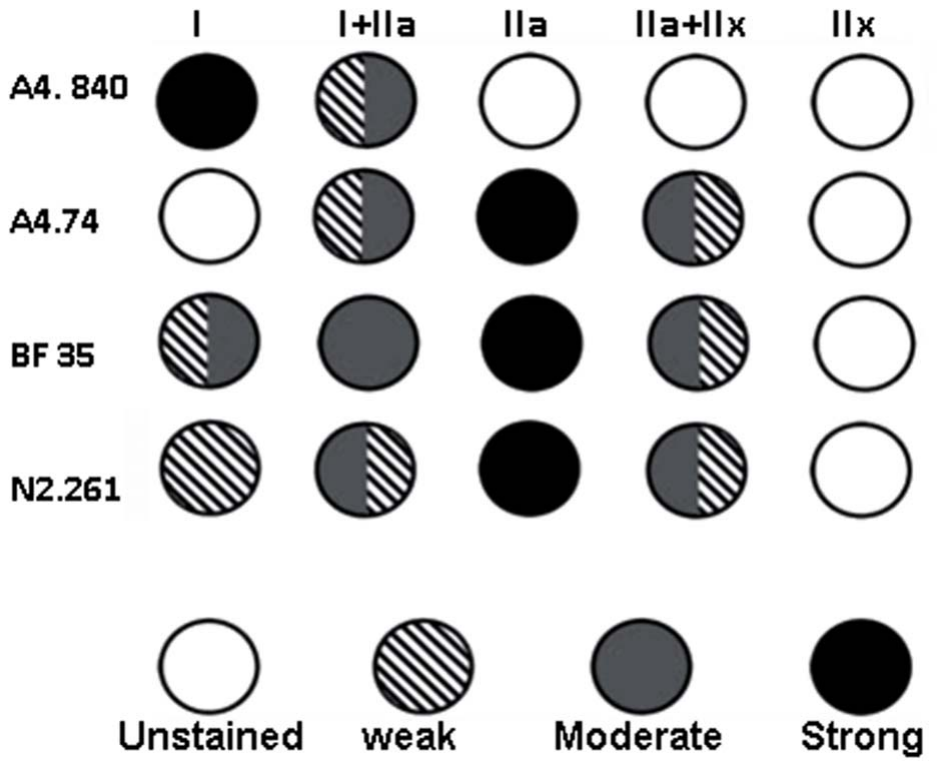
Scheme of fibre phenotype classification. The staining intensity of the four different antibodies in all fibres were briefly classified into “unstained”, “weak”, “Moderate” and “Strong”. An individual fibre was classified into a specific fibre type according to its staining intensities with the four different antibodies against different MyHC isoforms.

Fibre type classification was based on the staining pattern of the fibres for the mAbs directed against different MyHC isoforms. According to a staining scheme of these antibodies (Fig. 1), the fibres were classified into fibre phenotypes containing solely slow MyHCI (type I), fast MyHCIIa (type IIA) or fast MyHCIIx (type IIX) or hybrid fibres co-expressing MyHCI+IIa (type I+IIA) or MyHCIIa+IIx (type IIAX).

The sections stained with antibodies F1.652 and NCL-MHCn were examined for detection of fibres containing developmental MyHCs. All positively stained fibres were counted and expressed as percentage of total fibres. For analysis of infiltration of inflammatory cells, the sections stained with mAb against CD68 were scanned thoroughly to identify positively stained monocytes/macrophages. The number of fibres with infiltration of monocytes/macrophages was counted and expressed as number of cells per mm^2^ muscle cross-section. Necrotic muscle fibres containing monocytes/macrophages were not included in the counting.

### Statistical Analysis

Statistical analyses were performed using the StatView program (SAS Company, Berkley, CA, USA). Data are presented as mean ± SD. Since no indication of non-normality was observed within each group, a *t*-test for unpaired data was used. Because the biopsies taken at 2–3 days and at 7–8 days were from the same group of subjects, data of the two groups was further analysed by using a paired sample *t*-test. Statistical significance was set at p<0.05.

## Results

### DOMS and Sarcolemma Injury

All exercised subjects experienced severe DOMS that reached peak values (7.8±1.4 for loaded and 7.7±1.7 for unloaded) between 1.5 to 2.5 days and returned to pre-exercise level by 6 days post-exercise. There was no significant difference in the mean values of DOMS between the loaded and unloaded soleus muscles at any time [14]. The dynamic changes of DOMS following the time course after the downstairs running have been published previously [14].

**Figure 2:**
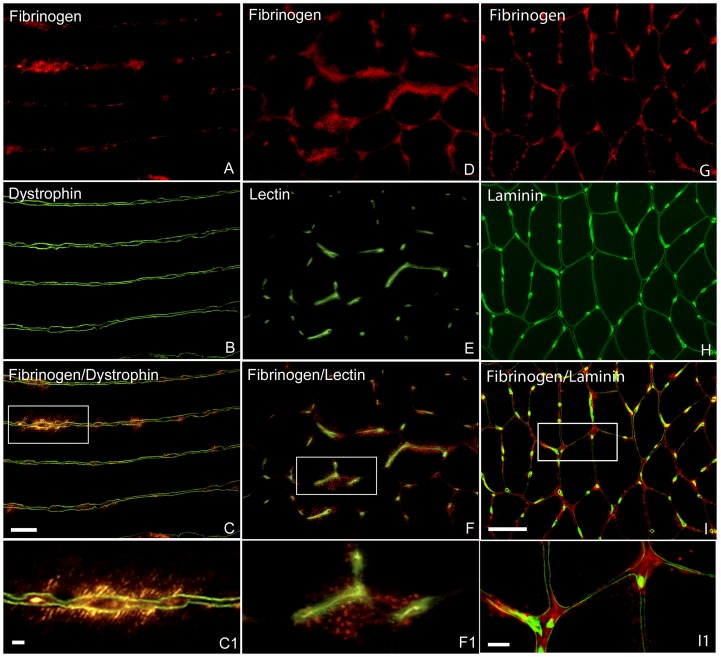
Immunofluorescence staining for visualization of muscle fibre sarcolemma integrity. **A–C**, one longitudinal section from a biopsy of 1 h group double stained for fibrinogen (**A**) and dystrophin (**B**). **C** is merged images of **A** and **B**, and **C1** is enlargement of boxed areas in **C**. Several sites of intracellular staining for fibrinogen outlined by dystrophin were seen along the length of a fibre, whereas staining for dystrophin did not reveal distinct disruption of plasma membrane. **D–F**, one transverse section from a biopsy of 1 h group double stained for fibrinogen (**D**) and lectin (**E**). F is merged images of **D** and **E**, and **F1** is enlargement of boxed areas in **F**. Intracellular staining for fibrinogen was seen close to capillaries revealed by lectin staining. **G–I**, one transverse section from a biopsy of control group double stained for fibrinogen (**G**) and laminin (**H**). **I** is merged images of **G** and **H**, and **I1** is enlargement of boxed areas in **I**. Several sites of distinct extracellular staining for fibrinogen outlined by laminin were seen. Bars 50 µm

No visible lesions were observed in muscle fibre sarcolemma proteins revealed by staining for dystrophin and laminin (Fig. 2). Staining for plasma fibronectin was negative in all muscle samples, except for one biopsy from the 1 h group, where a few fibres contained some small and weakly stained subsarcolemmal dots (not shown). In the same fibres, where intracellular staining for fibronectin was observed, similar subsarcolemmal dots revealed by double staining for fibrinogen were present as well. However, intracellular staining for fibrinogen was more often observed in fibres where staining for fibronectin was negative. The intracellular staining for fibrinogen was usually seen as a rim adjacent to the plasmalemma close to capillaries and exhibited a diminishing gradient towards the centre of the fibres (Fig. 2). The intracellular staining for fibrinogen was observed in a low proportion of fibres (<5%) in both the controls and exercised groups. Statistical analysis of the number of fibres with intracellular staining for fibrinogen did not reveal any significant difference between control and any post exercise groups.

In the samples of experimental myocardial infarction, all the three antibodies against fibrinogen showed distinct intracellular staining in necrotic cardiomyocytes as previously shown [38]. However, in the soleus muscle samples, positive staining for fibrinogen as small dots was seen in some areas in both muscle fibres and extracellular matrix (Fig. 2).

### Necrotic Fibre and Infiltration of Inflammatory Cells

**Figure 3:**
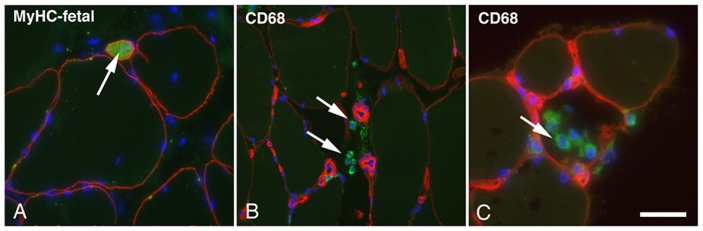
Immunofluorescence staining for visualization of developmental and inflammatory cells. **A**, one transverse sections from biopsy of 7–8 day group double stained with antibodies against MyHC-fetal (green) and dystrophin (red). One small neonatal fibre was seen between normal sized fibres (arrow). **B** and **C**, transvers sections from biopsies of 2–3 day group double stained with antibodies against CD68 (green) and laminin (red). Several extracellular monocytes/macrophages (arrows) were seen in **B** whereas in **C** monocytes/macrophages were seen inside a necrotic fiber. Nuclei (blue) were visualized in all sections by using mounting medium containing DAPI. Bar 100 µm

Routine histological staining showed that the fibres in both the controls and exercised samples had generally a polygonal shape and were tightly packed in well-ordered fascicles. A few monocytes/macrophages revealed by antibody against CD68 were seen in between fibres in both control and post exercise groups (Fig. 3). No significant difference was observed in the number of extracellular monocytes/macrophages per unit muscle cross-sectional area (CSA) between control (5.3±3.3 per µm^2^) and post exercise groups (1 h, 6.0±1.7 per µm^2^; 2–3 day, 11.9±3.3 per µm^2^; 7–8 day, 5.3±3.5 per µm^2^). Intracellular staining for CD68 was only observed in a few fibres that appeared to be necrotic (Fig. 3). Cells with intracellular staining for CD68 were not included in the data counting.

### Developmental MyHCs

A few fibres expressing developmental MyHCs were observed in the 2–3 day and 7–8 day groups, but not in the control or 1 h group (Fig. 3). These fibres were generally extremely small in size in the 2–3 day group (<100 µm^2^) and somewhat larger (<400 µm^2^) in the 7–8 day group.

### Muscle Fibre Phenotype Composition

The staining pattern for the different MyHC mAbs distinguished fibres containing MyHCI and MyHCIIa in all subjects and nearly all samples contained a small proportion of hybrid fibres co-expressing MyHCI+IIa or MyHCIIa+IIx. The control soleus muscle was characterized by a very high proportion of fibres containing slow MyHCI (68%±9) and a population of fibres containing fast MyHCIIa (26%±8). No significant difference in proportion of the different fibre phenotypes was observed between any group, except that one subject contained a low proportion of fibres expressing MyHCIIx at 2–3 days (1%±4) and 7–8 days (2%±4).

### Muscle Fibre Size

**Figure 4:**
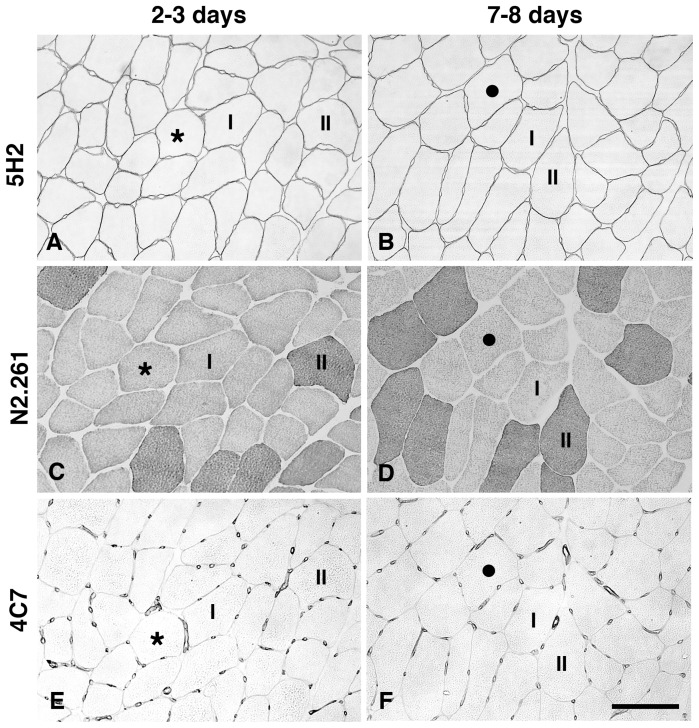
Immunohistochemical staining for visualization of muscle fibre size, fibre phenotype and capillary supply. Serial transverse sections from one biopsy in the 2–3 day group **(A, C, E)** and one from the 7–8 day group **(B, D, F)** stained with antibodies 5H2 for fibre profile **(A, B)**, N2.261 for fibre phenotypes **(C, D)** and 4C7 for capillaries **(E, F)**. Most fibres are larger in size in the 7–8 days group **(B)** compared to the 2–3 days group **(A)**. Type I and type II fibres are marked **(C, D)**. There was no distinct difference in number of capillaries around each fibre between the two groups **(E, F)** despite the fibre size was larger in the 7–8 day group **(F)**. Stars and black dots mark same fibre in series sections. Bar 100 µm

**Figure 5:**
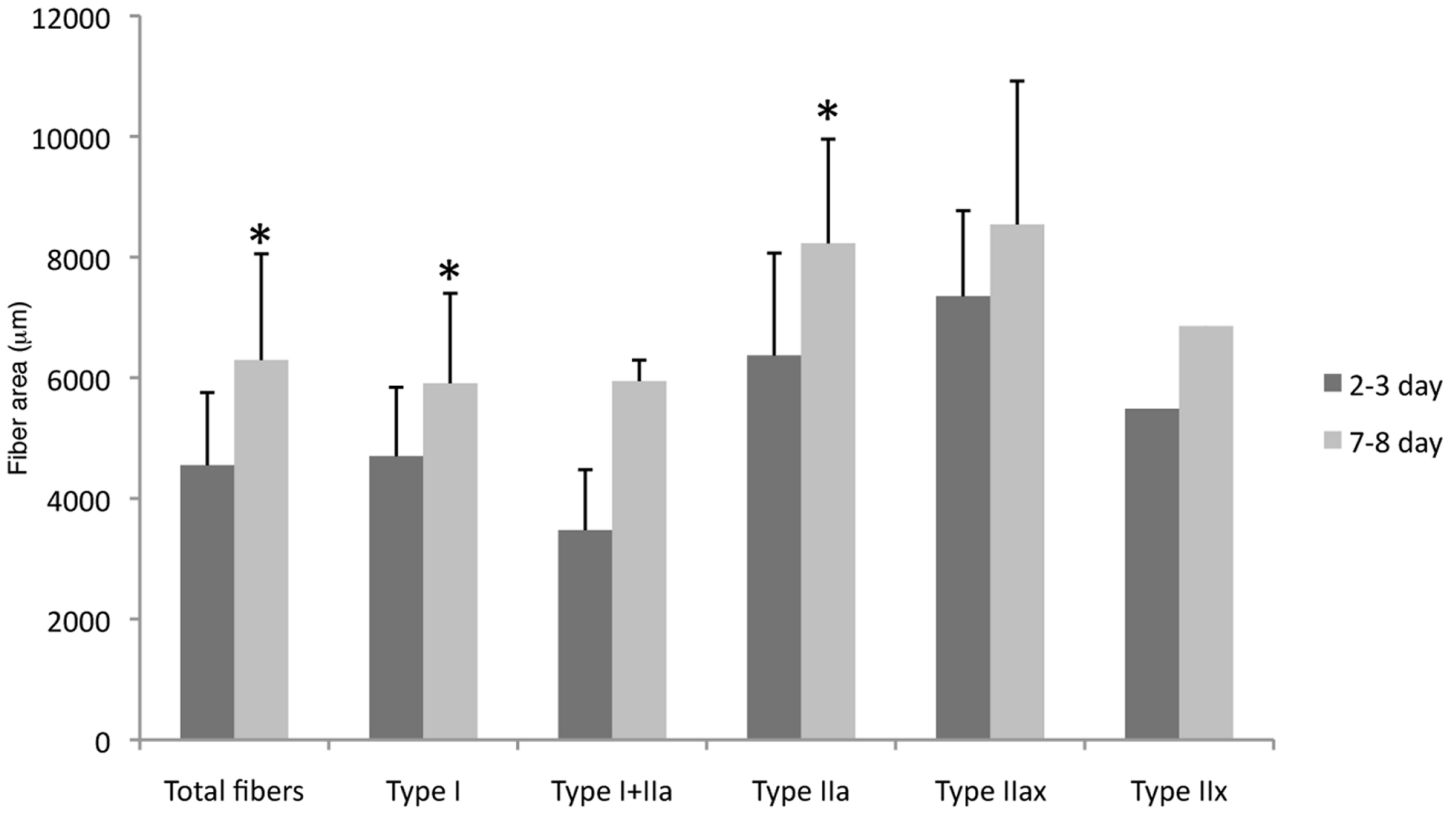
Comparison in fibre size between 2–3 day and 7–8 day groups. Fibre size in 7–8 day group was significantly larger than in 2–3 day group. Among the five different fibre phenotypes, only type I and type IIa fibres presented larger fibre size in 7–8 day group than in 2–3 day group (*p<0.05).

The mean fibre size was 5894 µm^2^±845 for the control group and 4930 µm^2^±1418 for the 1 h group, 4987 µm^2^±1367 for the 2–3 day group and 6588 µm^2^±1551 for the 7–8 day group. The fibre size at 7–8 days was significantly larger than that at 2–3 days (p<0.05; Fig. 4). Analysis of fibre size with respect to fibre phenotype revealed that type I and type IIa fibres in the 7–8 day group were significantly larger than in the 2–3 day group (Fig. 5). Variability in fibre size, expressed as CV, was approximately in the same range at 2–3 days and at 7–8 days (34% vs. 30%).

### Capillary Supply

**Figure 6:**
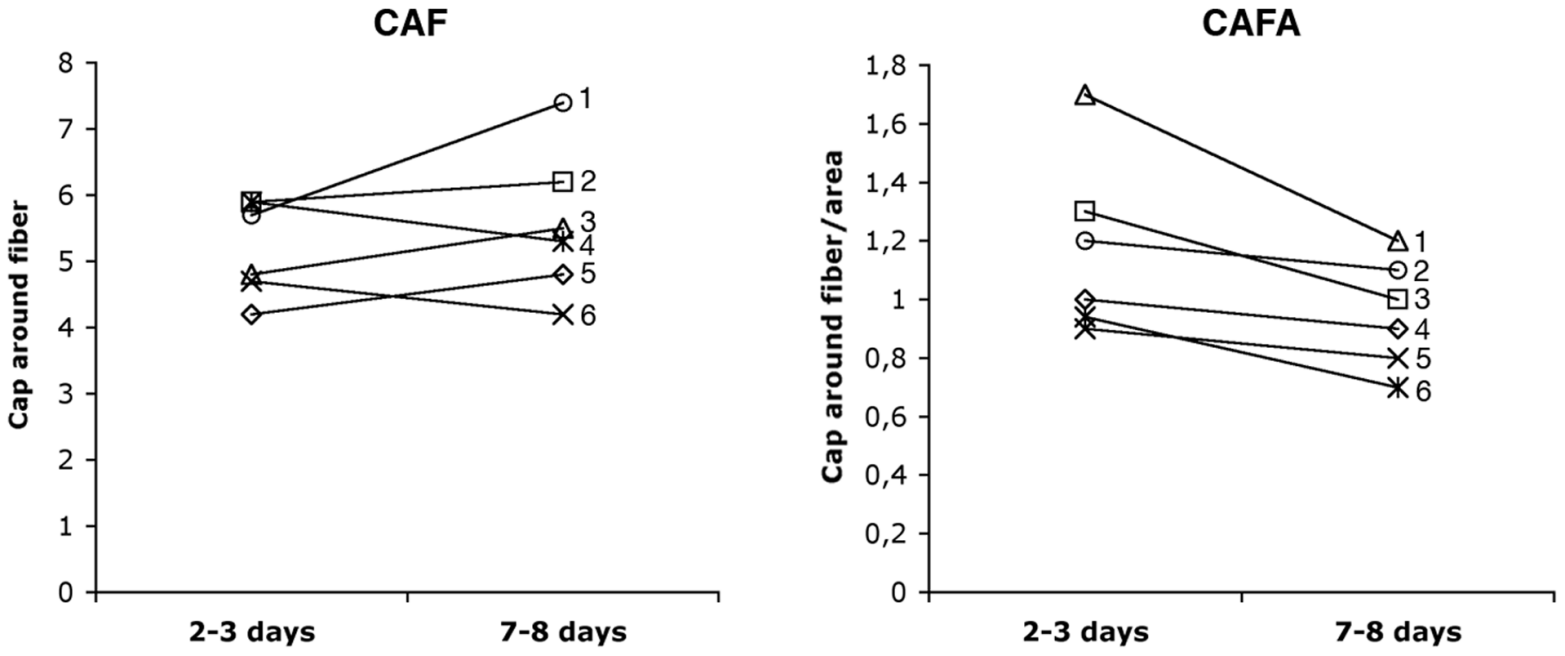
Comparison of individual values in CAF and CAFA from the same subjects in 2–3 day and 7–8 day groups. Compared to 2–3 day group, CAF was higher in three subjects (1, 3, 5) and lower in two subjects (4, 6), whereas CAFA was lower in five subjects (1, 3, 4, 5, 6) in 7–8 day group (p<0.05).

At the group level no significant difference in capillary parameters was observed between the control and the post-exercise groups, although a decreasing trend in capillary density (CD) was observed from 2–3 days to 7–8 days (330±6.1 vs. 289±71 cap/mm^2^; p = 0.07). Paired analysis of the subjects in the 2–3 day and 7–8 day groups revealed a significant difference in CAF in 5 of the 6 subjects, where 3 subjects had higher values and 2 subjects had lower values at 7–8 days than at 2–3 days (p<0.05; Fig. 6). When CAF was further processed by fibre size (CAFA), paired comparison showed lower CAFA value in 5 of the 6 subjects at 7–8 days compared to 2–3 days (p<0.05; Fig. 6).

## Discussion

The present study shows that the downstairs running leading to DOMS did not result in significant sarcolemma injury or serious inflammatory reactions in the soleus muscle. Thus, the hypothesis that eccentric exercise induces initial sarcolemma injury which triggers subsequent muscle fiber inflammation and necrosis seems not applicable for the human soleus muscle. The enlarged fiber size at 7–8 days post exercise was interpreted to represent fibre swelling due to edema. Since fiber swelling did not appear at the time of maximal DOMS (between 1.5 to 2.5 days post exercise), it seems not to be directly associated with the aetiology of DOMS.

### Sarcolemma Injury

Previous studies have mainly used two different methods to evaluate sarcolemma integrity after eccentric exercise: direct visualization through examination of muscle biopsy and indirect evaluation by measuring serum levels of muscle enzymes such as creatine kinase (CK), lactate dehydrogenase (LDH) and glutamic oxaloacetic transaminase (GOT), and of muscle proteins like slow myosin heavy chain (MyHC) fragments and myoglobin [1].

Many studies using animal models of eccentric muscular contraction have reported large proportions (around 10–30%) of muscle fibres with discontinuous or complete disruption of sarcolemma as evidenced by lack of staining for dystrophin, dystroglycans and sarcoglycans [43], [44] and by intracellular staining for serum albumin, fibronectin and Evans blue [19], [43], [44], [45]. In comparison, voluntary eccentric exercise of human muscles usually does not induce similar serious injury in sarcolemma integrity. In an early study on humans [10], ultra-marathon running (mainly downhill) induced very few fibres (in gastrocnemius muscle) with sarcolemma disruption. Using isolated high intensity eccentric exercise, two studies reported sarcolemma injury (in vastus lateralis muscle [46] and in biceps brachii muscle [47]), but the injury was only limited in a few of the subjects/biopsies (1 out of 8 subjects or 2 out of 24 biopsies [46], and 9 out of 61 biopsies [47]). Similarly, in a recent study, sarcolemma injury was only observed in 1 out of 18 biopsies (or 1 out of 9 subjects) from vastus lateralis muscle taken during (at 3 days) and after 6 days maximum voluntary eccentric exercise of knee joint [23]. The present study did not observe any significant lesion revealed by either sarcolemma proteins or by plasma proteins in any of the post exercise groups. The results of the study indicate that human soleus muscle sarcolemma was not as susceptible to the downstairs running as animal muscles to isolated eccentric exercise [18], [19], [43], [44], [45]. Importantly, the study did not observe sarcolemma damage at 1 hour after the downstairs running, indicating the previous hypothesis that early sarcolemma injury triggers subsequent deleterious reactions in muscles after eccentric exercise is not applicable to the human soleus muscles. The results of all these studies collectively indicate that sarcolemma disruption occurs in a minority of exercised subjects irrespective of how the voluntary eccentric exercise is performed.

Previous study has shown that disruptions of cell membranes are common in vivo and that the disrupted membranes can reseal within a minute even when a hole is up to 1 µm in diameter [48]. It is commonly believed that structural changes in muscles induced by eccentric exercise can be regarded as a continuum from mild changes to pathological-like changes [1]. We propose that the sarcolemma injuries in humans induced by voluntary eccentric exercise are mostly from mild to moderate in extent and therefore most injuries are temporary and reversible. The temporary injury in sarcolemma may allow the efflux of muscle enzymes such as CK, LDH and GOT, and even large molecular proteins like MyHC fragments and myoglobin into the plasma. Since such sarcolemma injuries are usually resealed very fast, no visible lesion in sarcolemma is available even if the level of muscle enzymes and muscle proteins are high in the serum. This might explain the poor correlation between serum CK level and changes in muscle histology [49].

In contrast, the electrical stimulation-induced eccentric muscular contractions might result in sarcolemma injuries too extensive in size to be resealed in animals (rabbit digitorum longus muscle [18] and rabbit and rat tibialis anterior muscle [18], [19]) and in humans (vastus lateralis muscle [5]). This might lead to calcium influx and subsequent proteinase activation and hydrolysis of structural proteins like desmin and sarcolemma proteins, followed by complete sarcolemma disruption and fibre necrosis [5], [18], [19]. Of course, sarcolemma damage to such an extent will allow efflux of muscle enzymes and muscle proteins, and even influx of serum proteins such as fibronectin, albumin and tetranectin [1], [5], [18], [19].

Sarcolemma injury in humans after voluntary eccentric exercise seems to be limited mainly in a few biopsies/subjects [10], [23], [46], [47], which indicates individual variation in sensitivity of muscle fibre sarcolemma to the same mode of eccentric exercise. This is consistent with the observation that post-exercise plasma CK activity demonstrates large variation across individuals with high responders, median responders and low responders [49]. Interestingly, some studies (including the present study) have shown that sarcolemma injury [10], [23], [46], [47] occurred often in the same biopsies/subjects where fibre necrosis were observed [5], [10], [46], [50], [51]. Currently, not much data is available about the relationship between sarcolemma injury and fibre necrosis after eccentric exercise; however, it seems plausible to speculate a close correlation between the two events, especially when sarcolemma injury is extensive.

The present study applied downstairs running to expose the soleus muscle to eccentric exercise. Compared with other studies using isolated maximal eccentric contraction on humans [5], [46], [47], [50], [51] and animals [17], [18], [19], the exercise mode used in the study might explain the lack of significant sarcolemma injury and inflammatory reaction.

Fibrinogen has been previously proved to be a good marker for cardiomyocyte damage after myocardial infarction [38]. However, in the present study, some small intracellular staining for fibrinogen existed not only in post exercise muscles (<5%) but also in controls (around 3.7%). In addition, in some areas, positive staining for fibrinogen was also present in extracellular matrix. As a control for the antibodies, the samples of the experimental myocardial infarction were also tested in the study, and all the three different antibodies against fibrinogen revealed only distinct intracellular staining in the necrotic cardiomyocytes. On basis of the results, we concluded that plasma fibrinogen might be a good marker for necrotic fibres, but not for detection of mild membrane damage.

### Necrotic Fibres and Infiltration of Inflammatory Cells

In the present study, very few necrotic fibres and a nearly negligible infiltration of monocytes/macrophages were observed in the exercised muscles. The findings were consistent with previous observations on humans after voluntary eccentric exercise [10], [20], [22], [30], [50], [51]. Studies on lower body muscles (vastus lateralis) failed to present fibre necrosis after high intensity of knee eccentric activity (30 min eccentric cycling [20], [22] and repetitive knee eccentric extension [30]). Even ultra-marathon footrace (160 km, mainly downhill) only resulted in very few necrotic fibres (1%) in gastrocnemius muscles [10]. Studies on upper body muscles (biceps brachii), however, indeed revealed fibre necrosis after maximal voluntary eccentric contractions of the elbow flexors, but it occurred only in a few of the exercise subjects/biopsies (3 out of 15 biopsies [50], and 8 out of 23 subjects [51]). These results seem in support of the observation that the upper body muscles are more prone to exercise-induced damage than lower body muscles [52].

In animal models of electrical stimulation-induced eccentric muscle contraction, massive muscular inflammation and necrotic fibres (over 30% necrotic fibres) were observed (in rabbit digitorum longus muscle [18] and in both rabbit and rat tibialis anterior muscle [18], [19]). In this context, the study of Crameri et al (5) is of special interest and relevance. Massive fibre necrosis was observed in vastus laterlis muscles subjected to electrical stimulation-induced eccentric muscle contractions whereas in the same subjects, no fibre necrosis was observed in the muscle of the contralateral leg after maximal voluntary eccentric contractions. The authors suggested that the difference in the extent of muscle injury between the voluntary eccentric exercise and the electrical stimulation-induced eccentric muscle contraction may be due to the difference in motor unit recruitment pattern. This may also explain the difference in the extent of fibre necrosis and inflammation between animals and humans. Alternatively, the difference in exercise protocol may play an important role in the extent of muscle injury.

### Developmental MyHCs

In the 2–3 day group, a few extremely small fibres expressed developmental MyHC. Such fibres were also observed in a very low number in the 7–8 day group, but these were relatively larger in size. In adult limb muscles, small fibres expressing developmental MyHC have been associated with newly formed/regenerated muscle fibres [53]. Satellite cells are considered to be involved in the process of repair and formation of new fibres after muscle damage [54]. Although we found no signs of serious muscle damage in the post exercise muscles, the findings of small fibres expressing developmental MyHCs in the post-exercise muscle biopsies may imply that the downstairs running induced a very restricted regenerative process in the soleus muscle.

### Muscle Fibre Swelling

Muscle fibre swelling due to oedema has been associated with the mechanisms of DOMS induced by eccentric exercise [55]. Muscle fibre swelling evaluated by increased muscle fiber size and intramuscular pressure has been observed 2 days post exercise in human tibialis anterior muscle after 400 submaximal eccentric contractions [25] and vastus lateralis muscle after repetitive maximal eccentric exercise [30]. However, since the two studies did not examine serial biopsies following the time course of DOMS after the eccentric exercise, the association between muscle fibre swelling and DOMS was still inconclusive. In the present study, we observed a 24% greater increase in muscle fibre size at 7–8 days than at 2–3 days in the same group subjects. The increased fibre size was interpreted to reflect muscle fibre swelling. However, a single bout of eccentric exercise has been shown to stimulate net protein synthesis within 4–8 hours after exercise [56]. Thus, we could not exclude the possibility that increased protein synthesis is partly responsible for the dramatic enlargement in fibre size at 7–8 days after the exercise. Yet, the results of the relatively similar fibre size in different fibre phenotypes and the significant lower CAFA values in 5 out of the 6 subjects at 7–8 days compared to 2–3 days suggest that the larger fibre size was most likely due to fibre swelling. Since fibre swelling appeared at 7–8 days, not at 2–3 days when DOMS peaked, we conclude that fibre swelling is not associated with the aetiology of DOMS in human soleus muscle after the downstairs running.

### Muscle Vascularization

In previous studies on rats, exhaustive eccentric exercise of spinotrapezius muscle [34] and 300 electrical stimulation-induced eccentric contractions of gastrocnemius muscles resulted in damage to microcirculation and thereby the delivery and exchange of O_2_ and substrates [35]. The damage was exhibited more often in the capillary lumen rather than capillary collapse or capillary degeneration. In this study, we did not observe significant alterations in capillary network at the group level. However, a significant increase in the capillary network was observed 7–8 days post exercise in some individuals, whereas a regression of the capillary network was found in others. The different responses to the downstairs running might reflect individual variation in physical fitness or inherent genetic differences. Although it is not possible to make any reliable conclusions about the effect of eccentric exercise on the capillary network, the present results indicate that the same exercise could lead to completely different reactions in the microvascular system in different subjects.

## Conclusions

The present study showed that the downstairs running did not induce early injury in sarcolemma or severe muscle fibre inflammation and necrosis. The result disproves the previous hypothesis that eccentric exercise leads to initial sarcolemma injury which triggers subsequent deleterious reactions such as muscle fibre inflammation and necrosis. The study revealed that the downstairs running indeed induced fibre swelling, but it appeared at 7–8 days, not at 2–3 days post exercise when DOMS peaked. The result also disproved previous hypothesis that muscle fibre swelling is directly associated with the symptom of DOMS. However, the study examined only the soleus muscles after downstairs running; thus, a definite conclusion cannot be made before evaluations of other muscles are performed in the same way. Furthermore, individual variation may confound the interpretation of the data since in the study only the 2–3 day group and the 7–8 day group are from the same subjects.
